# Cost-effectiveness analysis of a multifactorial fall prevention intervention in older home care clients at risk for falling

**DOI:** 10.1186/s12877-017-0599-9

**Published:** 2017-09-01

**Authors:** Wanrudee Isaranuwatchai, Johnna Perdrizet, Maureen Markle-Reid, Jeffrey S. Hoch

**Affiliations:** 1 Centre for Excellence in Economic Analysis Research, Li Ka Shing Knowledge Institute, St. Michael’s Hospital, 30 Bond Street, Toronto, ON M5B 1W8 Canada; 2 Institute of Health Policy, Management and Evaluation, University of Toronto, 155 College St, Toronto, ON M5T 3M6 Canada; 3 School of Nursing; Clinical Epidemiology and Biostatistics, McMaster University, 1200 Main St. W, Hamilton, ON L8N 3Z5 Canada; 4 Division of Health Policy and Management, Department of Public Health Sciences, University of California, Davis, 2103 Stockton Blvd. Sacramento, California, 95817 USA

**Keywords:** Cost-effectiveness analysis, Fall prevention, Age, Older adults, Multifactorial intervention

## Abstract

**Background:**

Falls among older adults can cause serious morbidity and pose economic burdens on society. Older age is a known risk factor for falls and age has been shown to influence the effectiveness of fall prevention programs. To our knowledge, no studies have explicitly investigated whether cost-effectiveness of a multifactorial fall prevention intervention (the intervention) is influenced by age. This economic evaluation explores: 1) the cost-effectiveness of a multifactorial fall prevention intervention compared to usual care for community-dwelling adults ≥ 75 years at risk of falling in Canada; and 2) the influence of age on the cost-effectiveness of the intervention.

**Methods:**

Net benefit regression was used to examine the cost-effectiveness of the intervention with willingness-to-pay values ranging from $0–$50,000. Effects were measured as change in the number of falls, from baseline to 6-month follow-up. Costs were measured using a societal perspective. The cost-effectiveness analysis was conducted for both the total sample and by age subgroups (75–84 and 85+ years).

**Results:**

For the total sample, the intervention was not economically attractive. However, the intervention was cost-effective at higher willingness-to-pay (WTP) (≥ $25,000) for adults 75–84 years and at lower WTP (< $5,000) for adults 85+ years.

**Conclusions:**

The cost-effectiveness of the intervention depends on age and decision makers' WTP to prevent falls. Understanding the influence of age on the cost-effectiveness of an intervention may help to target resources to those who benefit most.

**Trial registration:**

Retrospectively registered. Clinicaltrials.gov identifier: NCT00463658 (18 April 2007).

## Background

Falls among older community-dwelling adults cause significant problems, and evidence suggests that most falls are predictable and preventable [1–5]. Falls are the leading cause of injury-related hospitalization for seniors; and the economic costs of falls are likely greater than decision-makers expect [6, 7]. In general, falls result from the interaction of multiple risk factors, many of which are preventable [8–11]. Fall prevention strategies incorporating multifactorial and interprofessional approaches, aimed at multiple risk factors contributing to falls, might be the most successful [12]. Studies have shown that multifactorial programs are as effective at reducing falls as single intervention programs [13].

Although the goals of a fall prevention program are to improve health outcomes including reducing fall risk factors, preventing falls and fall-related use of health services, and maintaining quality of life, cost-effectiveness is also a critical factor. Decision- and policy-makers often have an implicit monetary value that they are willing to spend in order to produce a particular health outcome (e.g., prevent a fall), referred to as willingness-to-pay (WTP). An intervention may be effective at reducing falls, but if its costs are prohibitively high, it may not be a practical option for implementation given the WTP. To adequately inform policy- and decision-makers, both effectiveness and cost-effectiveness of interventions (including the suitability of intervention to individuals) need to be assessed.

Many fall prevention interventions for community-dwelling older adults have proven cost-effectiveness [3, 14–22]. However, similar interventions demonstrate conflicting cost-effectiveness results in different populations and settings [20, 23–29]. For example, some studies reported that multifactorial programs are an economically attractive option [17, 20, 22–26, 29] for reducing falls in community-dwelling older adults, whereas others studies reported the opposite [14, 15, 17]. These data suggest that multifactorial programs were not cost-effective and subsequently performed subgroup analysis, found the program was cost-effective for specific geographic regions or for particular community-dwelling subgroups [22, 24]. Therefore, the cost-effectiveness of a multifactorial program can vary depending on characteristics of community-dwelling older adults.

It is well established that fall incidence rates and related injuries steadily increase as a function of age [4]. Given that age is a significant risk factor for falls and fall-related injuries, multifactorial approaches could be more effective, and potentially cost-effective, if age was a factor considered in fall prevention programs. Markle-Reid et al. (2010a) conducted a randomized controlled trial (RCT) of a multifactorial intervention among community-dwelling older adults at risk of falling and found that compared to usual home care, the intervention was effective in reducing falls in 75–84 year old adults but not for 85+ year old adults. These findings are consistent with the idea that age is associated with the effectiveness of an intervention. Moreover, Wu et al. (2010) found that a multifactorial program was more cost-effective for younger (65–74 years) compared to older (≥75 years) adults [17]. Consequently, age has been shown to be a factor influencing the cost-effectiveness of a multifactorial fall prevention intervention [9]. However, the model by Wu et al. (2010) did not examine the variability of cost-effectiveness among older adults (≥75 years), who are at highest risk of falls.

Using cost and effectiveness data from Markle-Reid et al. (2010b), we conducted a secondary data analysis: 1) to determine the cost-effectiveness of a multifactorial fall prevention intervention compared to usual home care for community-dwelling older adults (≥ 75 years), and at risk of falling; and 2) to examine the influence of age on the cost-effectiveness of the intervention.

## Methods

### Study design

A cost-effectiveness analysis of a multifactorial and interprofessional fall prevention intervention (the intervention) was performed using cost and effectiveness RCT data from Markle-Reid et al. (2010) [30]. A total of 109 participants were randomly assigned to the intervention or usual home care services (usual care). The total analytical sample included 92 participants that completed the 6-month follow-up. Two age subgroups were explored: young-old (75–84 years) and old-old (85+ years).

### Setting and participants

Participants were community-dwelling older adults (≥75 years), referred to and eligible for personal support services through a Community Care Access Centre (CCAC) in Southern Ontario, mentally competent, and English-speaking. Individuals were classified as being at high-risk for falls if they answered “yes” to any of the following questions: 1) have you fallen in the past 12 months; 2) do you have a fear of falling; or 3) are you unsteady on your feet [9, 31]?

### Intervention

Usual care participants received home care services arranged by the CCAC care coordinator, which included follow-up assessments for in-home health services eligibility; arranging professional (i.e., nursing, occupational therapy, physiotherapy, social work, speech-language pathology, and nutrition) and non-professional services (i.e., personal support services and homemaking services)﻿; providing information and referral to community agencies; and monitoring care through in-home assessments. The intervention group received usual care, plus monthly in-home visits by an interprofessional team (CCAC case manager, registered nurse, occupational therapist, physiotherapist, and registered dietitian), with specialized training in the area of fall prevention. The interprofessional team’s goal was to prevent falls and fall-related injuries, enhance health and quality of life, and reduce use of health care services. Activities included: 1) conducting routine systematic assessments using standardized screening tools that identified risk factors for falls and poor health; 2) managing modifiable risk factors; 3) providing support; and 4) educating clients about fall prevention. The team met monthly to develop an interprofessional plan of care, and tailored the frequency and timing of the home visits to meet individual client needs. A detailed description of the intervention is described elsewhere [9, 30].

### Variables and measures

The outcome was the number of falls at six-months, measured by self-report. A fall was defined as unintentionally coming to rest on the ground or floor. Participants kept a calendar to record daily fall status. Interviewers blinded to treatment assignment telephoned participants monthly to obtain additional information on fall incidents recorded, using the Falls Surveillance Report, created by the research team [30].

The costs of health service utilization were measured using the Health and Social Services Utilization Inventory (HSSUI), which assesses costs from a societal perspective and has established reliability and validity [32–34]. The total cost per person was calculated by multiplying the number of units of service (quantity) and unit cost (price). The HSSUI consists of six direct health care service categories: 1) primary care; 2) emergency department and specialists; 3) hospital days; 4) other health and social care professionals; 5) prescription medications; and 6) lab services. Data collection was restricted to the reliable duration of recall: six months for hospitalization, two weeks for physician visits, and two days for prescription medication [32]. Questions to assess participant out-of-pocket costs are also included in the HSSUI, and described elsewhere [32]. Costs were reported in 2006 Canadian dollars (CAD). Cost and effect variables were defined as the *change* in falls or costs from baseline to follow-up. Baseline costs and effects were recorded for six-months *before* randomization. Follow-up costs and effects were recorded for six months *after* randomization. Potential confounders were identified from the literature [1, 5, 9, 30], including age, sex, fear of falling, and previous fall in the last six months.

### Statistical analysis

Analysis was performed using Stata (version 14, Stata Corp, Texas, USA). Student’s t-test was used for continuous variables and Chi-square test for categorical variables to compare characteristics between the intervention and usual care groups. We conducted two analyses: 1) for the total sample; and 2) by age subgroups (young-old defined as 75–84 year old adults and old-old defined as 85+ year old adults). The total sample analysis included all covariates, but the subgroup analyses omitted the age variable. Cost-effectiveness analysis was conducted using a net benefit regression (NBR) framework [35]. Net benefit (NB) values for each participant were calculated for six WTP values, varying from 0 to 50,000 CAD [35]. The WTP quantifies the value decision makers would be willing to pay to prevent one fall [36]. The NBR approach used a linear regression framework to facilitate the cost-effectiveness analysis. As each participant has a specific NB value for each WTP, we created a separate regression model for each WTP with a NB value as the dependent variable; the intervention variable was the primary independent variable, and the covariates were age, sex, fear of falling, and previous history of falling. From the regression findings, the coefficient estimate on the intervention variable represented the incremental net benefit (INB), given the specific WTP value used for that regression. A positive INB (INB > 0) indicated that the intervention was cost-effective; whereas a negative INB (INB < 0) demonstrated the opposite. The INB of the intervention compared to usual care was calculated for the total sample and, subsequently, by age subgroups. Cost-effectiveness acceptability curves (CEACs) were created for the total sample and age subgroups. The CEAC illustrates uncertainty and is a form of sensitivity analysis [37]. Each CEAC indicated the probability that the intervention was cost-effective compared to usual care for a given WTP value [38].

## Results

Table 1 compares participant characteristics between the intervention and usual care group for the 92 participants who completed the 6-month follow-up, young-old (*n* = 49), and old-old (*n* = 43). The number of falls decreased in both groups, from baseline to 6-month follow-up; however, there was no difference in the change in the mean number of falls, from baseline to 6-month follow-up, for both the intervention and usual care group (﻿mean change in falls ± standard deviation (SD): 0.3 ± 2.6 versus 0.3 ± 3.3, respectively). In the young-old subgroup, a greater reduction in the number of falls was found for the intervention (mean change in falls ± SD: 0.1 ± 1.7) compared to usual care (mean change in falls ± SD: −0.8 ± 2.4). In addition, there was a difference in the mean cost at baseline between the intervention (mean change in CAD ± SD: 17,533 ± 18,335) and usual care groups (mean change in CAD ± SD: 37,615 ± 32,833), *p* < 0.05. The change in mean cost from baseline to 6-month follow-up between the intervention and the usual care group was also different for the young-old group, *p* < 0.05 (mean change in CAD ± SD: -12,743 ± 17,498 vs. ﻿mean change in CAD ± SD: -32,056 ± 32,204).

**Table 1:** Participants’ characteristics for the total sample and by age subgroups

	Total sample, all ages		Young-old group, 75–84 years		Old-old group, 85–95 years	
Participant characteristic	Intervention (*n* = 49)	Usual care (*n* = 43)	Intervention (*n* = 27)	Usual care (*n* = 22)	Intervention (*n* = 22)	Usual care (*n* = 21)
Baseline mean number of falls ± SD	−1.8 ± 2.7	−1.7 ± 3.7	−1.1 ± 1.0	−1.0 ± 1.1	−2.6 ± 3.8	−2.3 ± 5.2
Follow-up mean number of falls ± SD	−1.4 ± 2.7	−1.3 ± 2.2	−0.9 ± 1.4	−1.8 ± 2.5	−2.1 ± 3.7	−0.8 ± 1.9
Change in mean number of falls ± SD^a^	0.3 ± 2.6	0.3 ± 3.3	0.1 ± 1.7	−0.8 ± 2.4	0.5 ± 3.4	1.5 ± 3.8
Baseline mean cost in CAD ± SD	20,154 ± 21,068	26,150 ± 28,132	17,533 ± 18335*	37,615 ± 32833*	23,372 ± 24,056	14,139 ± 15,298
Follow-up mean cost in CAD ± SD	5126 ± 3914	4800 ± 4305	4789 ± 3988	5559 ± 5359	5540 ± 3873	4004 ± 2733
Change in mean cost in CAD ± SD^a^	−15,028 ± 20,518	−21,350 ± 27,359	−12,743 ± 17498*	−32,056 ± 32204*	−17,831 ± 23,837	−10,135 ± 14,992
Mean age in years ± SD	84.1 ± 5.0	83.2 ± 5.1	80.5 ± 2.8	78.9 ± 2.5	88.6 ± 3.0	87.7 ± 2.7
Female (%)	67%	77%	70%	73%	64%	81%
Fear of falling (%)	41%	49%	41%	45%	41%	52%
Fall in the last 6 months (%)	73%	67%	67%	77%	82%	57%

^a^As fall is a bad outcome, we have added a negative sign to indicate a bad effect. Change refers to the difference between six-month follow-up and baseline. For mean change in number of falls, a high estimate implies more falls prevented. For change in mean number of falls, a positive estimate means that there were fewer falls in the “Follow-up” period than in the “Baseline” period. For change in mean cost, a lower estimate indicates fewer resources used

Intervention = multifactorial fall prevention intervention; Usual care = usual home care services; n = number of participants; SD = standard deviation. * denotes *p* < 0.05 using Welch’s *t*-test

### Cost-effectiveness

Table 2 reports cost-effectiveness results for the total sample at multiple WTP values. All INB values for each model were negative for the total sample and became more negative at higher WTP values. For example, the INB in CAD was -$5518 at a WTP of $1000 CAD and -$7359 at $25,000 CAD. Thus, for the total sample, the multifactorial fall prevention intervention was not cost-effective compared to usual care, regardless of a decision-maker’s WTP to prevent falls. After stratification, for the young-old group, the intervention was cost-effective for young-old with a WTP to prevent one fall of at least $25,000 CAD to prevent falls. Conversely, for the old-old group, the intervention was cost-effective at WTP < $5000 CAD to prevent falls.

**Table 2:** Incremental net benefits for six willingness-to-pay values to prevent a fall for the total sample and by age subgroups

	Total sample, all ages (*n* = 92)^a^		Young-old group, 75–84 years (*n* = 49)^b^		Old-old group, 85–95 years (*n* = 43)^b^	
WTP	INB of intervention (CAD) [95% CI]	*P*	INB of intervention (CAD) [95% CI]	*P*	INB of intervention (CAD) [95% CI]	*P*
WTP = 0 CAD	−5441 [−15,622 to 4739]	0.291	−20,766 [−35,144 to −6388]	0.006	7739 [−5384 to 20,861]	0.240
WTP = 1000 CAD	−5518 [−15,825 to 4789]	0.290	−19,816 [−34,355 to −5277]	0.009	6190 [−7235 to 19,614]	0.356
WTP = 5000 CAD	−5825 [−17,909 to 6259]	0.341	−16,015 [−31,981 to −49]	0.049	−6 [−17,845 to 17,834]	0.999
WTP = 10,000 CAD	−6208 [−22,389 to 9972]	0.448	−11,264 [−30,383 to 7855]	0.241	−7750 [−34,753 to 19,253]	0.565
WTP = 25,000 CAD	−7359 [−39,756 to 25,039]	0.653	2989 [−29,753 to 35,730]	0.855	−30,983 [−90,576 to 28,611]	0.299
WTP = 50,000 CAD	−9276 [−71,168 to 52,616]	0.766	26,743 [−32,402 to 85,888]	0.367	−69,704 [−186,121 to 46,713]	0.233

^a^All six NB regression models for the full sample were adjusted for age, sex, fear of falling, and previous history of falling

^b^All six NB models for age subgroup analysis were adjusted for sex, fear of falling, and previous history of falling

A positive INB indicates that the intervention was cost-effective when compared to usual care

*INB* incremental net benefit, *Intervention* multifactorial fall prevention intervention; *CI* confidence interval, *P p*-value, *WTP* willingness-to-pay

### Sensitivity analysis

**Fig. 1:**
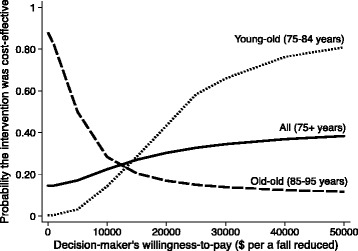
A cost-effectiveness acceptability curve indicates the probability that the intervention is cost-effective compared to usual care for a given willingness-to-pay value. The y-axis represents the probability that the intervention was cost-effective, and the x-axis represents a range of willingness-to-pay values. Cost-effectiveness acceptability curves for all (75+ years), young-old (75-84 years), and old-old (85-95 years)

The probability that the intervention was cost effective for the total sample, young-old, and old-old subgroups is shown in Fig. 1. When age was not considered, the probability that the intervention was cost-effective ranged from 15% to 38% across WTP values (up to 50,000 CAD). However, when age was stratified by subgroups, the probability that the intervention was cost-effective was 1% in young-old and 82% in old-old at a WTP of $1000 CAD. The opposite trend was found at higher WTP values. For example, with a WTP of 25,000 CAD to prevent a fall, the probability that the intervention was cost-effectiveness increased to 58% in young-old and decreased to 15% in the old-old group.

## Discussion

Cost-effectiveness of the multifactorial fall prevention intervention depended on age and decision-makers’ WTP to prevent a fall. With a specific WTP in mind, decision-makers could target those most likely to benefit from a fall prevention intervention in community-dwelling adults at risk of falling. To our knowledge, this is the first study to examine the influence of age on the cost-effectiveness of a multifactorial fall prevention intervention, and to provide the cost-effective results over a range of ages and WTP values.

Age influenced the cost-effectiveness of the intervention. With a small incremental benefit, the intervention was not economically attractive for the total sample. However, it was cost-effective for adults 85+ years at WTP values (< $5000 CAD) and for adults 75–84 years at higher WTP values (≥ $25,000 CAD) to prevent a fall. As the findings for different age subgroups differed, the WTP value and subsequently the intervention’s cost-effectiveness could differ for different subgroups as well. Recognizing the incremental benefits of the intervention, further research should explore which components of the multifactorial intervention benefit most in different age subgroups in order to optimize resource allocation and achieve value for each age subgroup.

Our study adds to the literature of the cost-effectiveness of multifactorial approaches for fall prevention in community dwelling older adults reporting mixed results [14, 15, 17, 20, 22–26, 29]. Beard et al. (2006) and Rizzo et al. (1996) reported a multifactorial program to be more cost-effective than usual care, and found cost-savings for the health care system that was attributed to the reduction in falls and fall-related injuries. Both studies incorporated an exercise component in the multifactorial intervention. However, Church et al. (2011, 2012) and Frick et al. (2010) found multifactorial approaches were dominated by several other fall prevention strategies in terms of cost-effectiveness for preventing falls, preventing hospitalizations, and more quality-adjusted life years (QALYs). Furthermore, Hendriks et al. (2008), Irvine et al. (2010), and Peeters et al. (2011) reported that the intervention was not cost-effective compared to usual care, which was replicated in our results using the total sample. Potentially, these studies could have found that the intervention was cost-effective for particular ages and WTP values if they were to explore age subgroups. Jenkyn et al. (2012) was the only study that used patient-level data to perform subgroup analysis, which demonstrated that a fall prevention program could be cost-effective for adults living in particular geographic regions in Ontario. Even so, conflicting findings regarding the cost-effectiveness of multifactorial programs in community dwelling adults could be due to differences in the intervention, study design, location, patient demographics, effective measures, unit costs, perspective, and time-horizon. This study reported that the cost-effectiveness of a multifactorial fall prevention intervention depended on an individual’s age and decision-makers’ WTP. The findings (e.g., impact of age on the cost-effectiveness of an intervention) may assist in the planning and optimizing resource allocation by directing services to those most likely to experience value.

Our study had a number of strengths. The sample from the original RCT was representative of the Canadian population at high risk of falls, since it was comprised of older adults using home services reporting fall risks [30]. The prevalence of falls in seniors with chronic needs and receiving home support services was about 70% [9, 30], which greatly exceeded the 30% fall rate among the general population of community-dwelling seniors reported in the literature [39–42]. The cost-effectiveness analysis used NBR, which allowed for: [1] controlling imbalances between intervention and usual care baseline characteristics remaining after randomization; [2] exploring subgroup analysis; [3] checking modeling assumptions; and [4] avoiding pitfalls associated with reporting negative incremental cost-effectiveness ratios. Moreover, CEACs conveyed uncertainty about the cost-effectiveness of the intervention [43].

Several limitations should be noted. The sample size was small (*n* = 92); nonetheless, the data came from a well-conducted RCT [9, 30]. Furthermore, the number of falls might have been underreported; since older people may fear loss of independent lifestyle and associated stigma of aging [44]. Nevertheless, given that study groups were randomized, underreporting should have been similar across groups. The outcome was the reduction in falls, which limited direct comparisons to studies using similar but slightly different outcomes (e.g., number of falls during the study). However, our outcome was appropriate since it took into account previous fall history and was based on its relevance to clinical practice. There were a few statistical limitations when examining the influence of age. By splitting the continuous age variable into subgroups for stratification, we lost precision in cost-effectiveness results. However, policy decisions are often based on artificial age groupings; thus, stratified results can be easily used by decision-makers.

## Conclusions

The multifactorial fall prevention intervention could be economically attractive depending on age and decision-makers’ WTP to prevent falls. The study contributed to the literature regarding the effect of age on the cost-effectiveness of multifactorial interventions; the intervention was cost-effective in certain contexts. Understanding the influence of age on the cost-effectiveness of an intervention may help to target resources more effectively to those most likely to benefit.

## Abbreviations

CAD: Canadian dollars
CCAC: Community Care Access Centre
CEACs: Cost-effectiveness acceptability curves
HSSUI: Health and Social Services Utilization Inventory
INB: Incremental net benefit
NB: Net benefit
NBR: Net benefit regression
QALYs: Quality-adjusted life years
RCT: Randomized controlled trial
SD: Standard deviation
WTP: Willingness-to-pay
